# New section on plant conservation genetics can help to achieve global conservation goals

**DOI:** 10.1002/ece3.10507

**Published:** 2023-09-05

**Authors:** Alison Gonçalves Nazareno

**Affiliations:** ^1^ Department of Genetics, Ecology and Evolution Federal University of Minas Gerais Belo Horizonte Brazil

**Keywords:** convention on biological diversity, population genetics, standing genetic variation

Since the establishment of a mathematical theory of population genetics by Ronald Fisher, JBS Haldane, and Sewall Wright, the field of population genetics has evolved significantly. Developments in the field were based on new theoretical frameworks (e.g., Moran and Cannings models [Cannings, 1974; Ewens, 2004; Freund, 2020], Ewens' sampling theory of neutral alleles [Ewens, 1972], Kimura's neutral theory [Kimura, 1983], and the Kingman coalescence theory [Kingman, 1982]), the improvement of molecular techniques to obtain empirical data (e.g., polymerase chain reaction, gel electrophoresis, Sanger's ‘chain‐termination’ sequencing technology, DNA microarray, and high‐throughput DNA sequencing technologies; Roalin, 2022), and advances in statistical and computational tools to analyze genetic data. These advances have enabled us to better understand the complex patterns of genetic variation and how microevolutionary processes, such as selection, migration, and mutation, shape standing genetic variation (i.e., genetic diversity). The ongoing advances and improvements in theoretical and empirical population genetics approaches (Charlesworth & Charlesworth, 2017), have allowed us to answer outstanding biological and evolutionary questions, as well as solve practical problems across a diverse array of fields. A straightforward corollary of the role played by advances in population genetics lies in the conservation and management of threatened species (Russello et al., 2020; Theissinger et al., 2023). Yet, there is still a gap between science and conservation policy.

Bringing genetic diversity to the forefront of conservation policy and management of threatened plant species is vital as the number of small and isolated forest fragments has increased dramatically over the last several decades, most notably in the tropics. For instance, considering an annual gross deforestation rate of 0.51%, there are 55.5 million small forest fragments on the American continent with a mean size of just 17.0 ha (Taubert et al., 2018). Consequently, a plethora of plant species are experiencing reductions in population size (Brummitt et al., 2015) and a loss of within‐population genetic diversity (Exposito‐Alonso et al., 2022; Leigh et al., 2019). Some of these species are highly susceptible to demographic stochasticity. Undoubtedly, this picture represents a serious threat to biodiversity at all levels, but particularly for genetic diversity since it has important ecological impacts on populations, communities, ecosystems, and significant evolutionary effects at the species level.

Due to its relevance, standing genetic variation is one of the biodiversity elements that the Convention on Biological Diversity (CBD) has advocated for in conservation and management policies (CBD, 2010). Launched on July 2021 and improved on December 2022, the Kunming‐Montreal biodiversity framework agreed at the UN Biodiversity Conference—the CBD's draft post‐2020 global biodiversity framework (GBF)—is an important strategic plan proposed to halt and reverse biodiversity loss for decades to come (GBF, https://www.cbd.int/doc/c/e6d3/cd1d/daf663719a03902a9b116c34/cop‐15‐l‐25‐en.pdf; CBD, 2022). Although genetic diversity goals and targets (specifically Goal A, Target 4, and those related to it) require further improvement (Hoban et al., 2023), most recent recommendations for the post‐2020 strategic plan for biodiversity give more significance to genetic indicators (e.g., the proportion of populations within species with an effective population size >500) and focuses on all species (https://www.cbd.int/doc/recommendations/wg2020‐05/wg2020‐05‐rec‐02‐en.pdf). In this context, assessing genetic diversity patterns, particularly for threatened plant species, is paramount to formulate effective policies for biodiversity conservation and design management programs.

In line with the post‐2020 GBF, Ecology and Evolution's new section on Plant Conservation Genetics seeks to encourage and support studies from around the world committed to restoring, protecting, maintaining, managing, and monitoring intra‐ and interpopulation genetic diversity. The journal's mandate to be author‐friendly, without the strict limitation of novelty, means the journal welcomes theoretical and practical population genetics studies that are engaged with the issue of plant conservation. Furthermore, the journal's mission is to help researchers involved in conservation genetics disseminate their work, so it can be used and applied in conservation and management programs not only by the scientific community but also by decision‐makers. Ecology and Evolution's editorial team hope that this new section will help the 196 parties to the CBD achieve global conservation goals and targets, specifically those related to safeguarding, restoring, and maintaining genetic diversity and adaptive potential within and among populations of all plant species. We encourage authors to include genetic diversity indicators (e.g., effective population size) in their plant conservation studies as recommended in the GBF (https://www.cbd.int/doc/decisions/cop‐15/cop‐15‐dec‐05‐en.pdf).

## AUTHOR CONTRIBUTIONS

**Alison Gonçalves Nazareno:** Conceptualization (equal); writing – original draft (equal).

## Data Availability

No data are available.
